# Alcohol Dehydrogenase Protects against Endoplasmic Reticulum Stress-Induced Myocardial Contractile Dysfunction via Attenuation of Oxidative Stress and Autophagy: Role of PTEN-Akt-mTOR Signaling

**DOI:** 10.1371/journal.pone.0147322

**Published:** 2016-01-25

**Authors:** Jiaojiao Pang, Nathan D. Fuller, Nan Hu, Linzi A. Barton, Jeremy M. Henion, Rui Guo, Yuguo Chen, Jun Ren

**Affiliations:** 1 Department of Emergency, Qilu Hospital of Shandong University, Jinan, Shandong, PR China; 2 Center for Cardiovascular Research and Alternative Medicine, University of Wyoming College of Health Sciences, Laramie, WY, United States of America; 3 Shanghai Institute of Cardiovascular Diseases, Zhongshan Hospital, Fudan University, Shanghai, PR China; University of Cincinnati, College of Medicine, UNITED STATES

## Abstract

**Background:**

The endoplasmic reticulum (ER) plays an essential role in ensuring proper folding of the newly synthesized proteins. Aberrant ER homeostasis triggers ER stress and development of cardiovascular diseases. ADH is involved in catalyzing ethanol to acetaldehyde although its role in cardiovascular diseases other than ethanol metabolism still remains elusive. This study was designed to examine the impact of ADH on ER stress-induced cardiac anomalies and underlying mechanisms involved using cardiac-specific overexpression of alcohol dehydrogenase (ADH).

**Methods:**

ADH and wild-type FVB mice were subjected to the ER stress inducer tunicamycin (1 mg/kg, i.p., for 48 hrs). Myocardial mechanical and intracellular Ca^2+^ properties, ER stress, autophagy and associated cell signaling molecules were evaluated.

**Results:**

ER stress compromised cardiac contractile function (evidenced as reduced fractional shortening, peak shortening, maximal velocity of shortening/relengthening, prolonged relengthening duration and impaired intracellular Ca^2+^ homeostasis), oxidative stress and upregulated autophagy (increased LC3B, Atg5, Atg7 and p62), along with dephosphorylation of PTEN, Akt and mTOR, all of which were attenuated by ADH. In vitro study revealed that ER stress-induced cardiomyocyte anomaly was abrogated by ADH overexpression or autophagy inhibition using 3-MA. Interestingly, the beneficial effect of ADH was obliterated by autophagy induction, inhibition of Akt and mTOR. ER stress also promoted phosphorylation of the stress signaling ERK and JNK, the effect of which was unaffected by ADH transgene.

**Conclusions:**

Taken together, these findings suggested that ADH protects against ER stress-induced cardiac anomalies possibly via attenuation of oxidative stress and PTEN/Akt/mTOR pathway-regulated autophagy.

## Introduction

Endoplasmic reticulum (ER), an intracellular membranous network, plays a crucial role in the maintenance of normal cardiac function through managing intracellular Ca^2+^ storage, folding and processing of proteins, lipid and sterol. External stimuli including hypoxia, glucose deprivation, oxidative stress, disorder of Ca^2+^ and infection may interrupt ER function [1], leading to a state of ER stress, which then activates unfolded protein response (UPR) [2]. Although UPR is a defense mechanism through a complex network to maintain ER homeostasis, excessive ER stress causes inappropriate activation of UPR, resulting in cell death through apoptosis or autophagy [3, 4]. A number of acute and chronic diseases including obesity [5], diabetes mellitus [6, 7], heart failure [8, 9], cardiac and cerebral ischemia/reperfusion injury [10, 11] have been consolidated with presence of ER stress, suggesting the therapeutic potential of targeting ER stress in these comorbidities. However, the mechanisms under ER stress-induced cardiac dysfunction have not been fully elucidated. Tunicamycin, a mixture of homologous nucleoside antibiotics, has been used as an ER stress inducer by blocking N-linked glycosylation of newly synthesized glycoproteins, thus resulting activation of UPR [12].

Alcohol dehydrogenase (ADH) is an essential enzyme catalyzing ethanol into acetaldehyde, which is then catalyzed by aldehyde dehydrogenase (ALDH2) to acetic acid. Five classes ADH have been identified in humans, with a prominent role for class I ADH in ethanol metabolism. Class I ADH consists of three subunits (α, β and γ) encoded by three genes (*ADH1A*, *ADH1B* and *ADH1C*) [13]. Although seven *ADH* genes are mapped for five classes of ADH, genetic polymorphism is only found for *ADH1B* and *ADH1C* genes. The mutant allele ADH1B*2 encodes a superactive ADH1B subunit. The enzyme encoded by ADH1B*1 /*1 displays only 1% and 0.5% of enzymatic activity of ADH1B*1/*2 and ADH1B*2/*2, respectively. The incidence of ADH1B*2 is much higher (40–90%) in East Asian populations compared with that in African and European populations (< 15% and 10%, respectively) [14, 15]. Two alleles exist for the *ADH1C* gene, namely ADH1C*1 and ADH1C*2, with a much greater catabolic efficacy to convert alcohol into acetaldehyde for ADH1C*1 allele as opposed to ADH1C*2 [14]. ADH polymorphism results in superactive ADH enzymatic activity to metabolize alcohol to acetaldehyde and increased risk of cardiovascular diseases. In myopathic alcoholic anomalies, ADH may worsen the unpleasant feeling of alcohol [16, 17]. Interestingly, class I ADH displays dismutatic property to catalyze endogenous aldehyde into both ethanol and acetate [18]. ADH is also capable of detoxifying lipid peroxidation products such as 4-hydroxyalcenals (4-HNE) [19]. ADH has been shown to exert protective effect against nonalcoholic diseases [20, 21]. Recent evidence has reported that ADH1B levels are inversely correlated with body mass index, waist circumference and fast plasma insulin level, suggesting its role as a novel candidate for obesity and insulin resistance [22]. Therefore, it is pertinent to examine the effect of ADH regardless of alcohol exposure on general health given the large population of polymorphism of ADH. To this end, this study was designed to evaluate the effect of ADH overexpression on ER stress-induced cardiac pathological changes using a cardiac-specific ADH overexpression mouse model.

## Methods and Materials

### Murine model of ADH cardiac-specific overexpression and tunicamycin treatment

All animal experimental procedures were conducted in accordance with the NIH Guide for the Care and Use of Laboratory Animals and were approved by the Animal Care and Use Committees at the University of Wyoming (Laramie, WY, USA) and Qilu Hospital of Shandong University (Jinan, Shandong, China). Production of the ADH transgenic mice was described in detail previously[16]. In brief, cDNA encoding murine class I ADH was inserted behind mouse α-myosin heavy chain promoter to achieve cardiac-specific overexpression in the albino Friendly Virus-B type (FVB) mice. Meanwhile, a cDNA encoding tyrosinase, an enzyme produces coat color pigmentation in albino mice, was co-injected with ADH to identify transgenic mice. All mice were housed in a temperature-controlled room under a 12 hr/12 hr-light/dark cycle and allowed access to tap water *ad libitum*. To induce ER stress, 4–6 month-old mice of both genders were delivered a single intraperitoneally injection of 1 mg/kg tunicamycin (Sigma-Aldrich, St. Louis, MO) dissolved in phosphate buffered saline (PBS) or an equivalent volume of PBS [23, 24]. All mice were used for experimentation 48 hrs after injection.

### Echocardiographic assessment

Cardiac geometry and function were evaluated in anesthetized [light (~1%) isoflurane inhalation] mice using the 2-dimensional (2-D) guided M-mode echocardiography (Phillips Sonos 5500, Amsterdam, Holland) equipped with a 15–6 MHz linear transducer. The heart was imaged in the 2-D mode in parasternal long-axis view with a depth setting of 2 cm. The M-mode cursor was positioned perpendicular to the interventricular septum and posterior wall of left ventricle (LV) at the level of papillary muscles from 2-D mode. LV anterior and posterior wall dimensions during diastole (LVPWd) and systole (LVPWs) were recorded from three consecutive cycles in M-mode using the method adopted by the American Society of Echocardiography. Fractional shortening was calculated from LV end-diastolic (LVEDD) and end-systolic (LVESD) diameters using the equation (LVEDD − LVESD)/LVEDD×100. Heart rates were averaged from 10 cardiac cycles [25].

### Isolation of cardiomyocytes

After ketamine sedation, hearts were rapidly removed from mice and mounted onto a temperature-controlled (37°C) Langendorff system. A modified Tyrode solution (Ca^2+^ free) is used to perfuse the hearts for 2 min, then the hearts were digested for 10–15 min with a modified Tyrode solution containing Liberase Blendzyme 4 (Hoffmann-La Roche Inc., Indianapolis, IN). The modified Tyrode solution (pH 7.4) contained the following (in mM): NaCl 135, KCl 4.0, MgCl_2_ 1.0, HEPES 10, NaH_2_PO_4_ 0.33, glucose 10, and butanedione monoxime 10, and the solution was gassed with 5% CO_2_−95% O_2_. The digested hearts were then removed from the cannula and the left ventricles were cut into small pieces in the modified Tyrode solution. Tissue pieces were gently agitated and pellet of cells was resuspended. Extracellular Ca^2+^ was added incrementally back to 1.20 mM over a period of 30 min. Isolated cardiomyocytes were used for the study within 8 h of isolation. Only rod-shaped cardiomyocytes with clear sarcomere striations were selected for mechanical studies [26, 27].

### Cell shortening/relengthening

Mechanical properties of cardiomyocytes were assessed using a SoftEdge Myocam (IonOptix, Milton, MA). Cardiomyocytes were placed in a chamber mounted on the stage of an inverted microscope (Olympus IX-70) and superfused (~2 ml/min at 25°C) with a Krebs-Henseleit bicarbonate buffer containing 1 mM CaCl_2_. Myocytes were field stimulated at 0.5 Hz. Cell shortening and relengthening were assessed including peak shortening (PS), indicating peak contractility; time-to-PS (TPS), indicating contraction duration; time-to-90% relengthening (TR_90_), indicating relaxation duration; and maximal velocities of shortening/relengthening (± dL/dt), indicating maximal pressure development and decline [28, 29]. To assess the role of autophagy, Akt and mTOR signaling in tunicamycin-induced cardiomyocyte contractile dysfunction, if any, cardiomyocytes from adult FVB or ADH transgenic mice were exposed to tunicamycin (3 μg/ml) for 4 hrs [24] in the presence or absence of the autophagy inhibitor 3-methyladenine (3-MA, 10 mM) [30], the Akt inhibitor AktI (1 μM) [31], or the mTOR inhibitor rapamycin (5 μM) [32] prior to mechanical assessment.

### Intracellular Ca^2+^ transient

A cohort of cardiomyocytes was loaded with fura-2/AM (0.5 μM) for 15 min, and fluorescence intensity was recorded with a dual-excitation fluorescence photomultiplier system (Ionoptix). Cardiomyocytes were placed onto an Olympus IX-70 inverted microscope and were imaged through a Fluor ×40 oil objective. Cardiomyocytes were exposed to light emitted by a 75-W lamp while being stimulated to contract at a frequency of 0.5Hz. Fluorescence emissions were detected between 480 and 520 nm; qualitative change in fura-2 fluorescence intensity (FFI) was inferred from the fura-2 fluorescence intensity ratio at the two wavelengths (360/380 nm). Fluorescence decay time (single exponential) was calculated as an indicator of intracellular Ca^2+^ clearance [33].

### Analysis of ROS production

Status of oxidative stress was measured using the ROS indicator 2',7'-dichlorodihydrofluorescein diacetate (H_2_DCFDA) or the O_2_^−^ detector dihydroethidium (DHE). For DCF staining, isolated cardiomyocytes from FVB or ADH mice were incubated with H_2_DCFDA (10 μM) for 1 hr at room temperature. Following 2 washes by PBS, isolated cardiomyocytes were treated with tunicamycin (3 μg/ml) or equivalent volume of DMSO for 2 hrs. After 3 rinses using PBS, cell fluorescence was measured using black 96 wells plates, at an excitation wavelength of 485 nm and an emission wavelength of 535 nm. Cardiomyocytes in each group were then lysed (same method as described in 2.7) and protein density was determined. The final fluorescence level was adjusted using protein content. For DHE staining, cardiomyocytes were first treated with DHE fluorescent dye (20 μM) for 1 hr at room temperature following tunicamycin treatment. DHE fluorescence was detected at an excitation wavelength of 520 nm and an emission wavelength of 595 nm. The rest of procedures were similar to that described for DCF [34, 35]. Representative images were taken using fluorescence microscope.

### Western blot analysis

Pellets of cardiomyocytes were sonicated in a lysis buffer containing 20 mM Tris (pH 7.4), 150 mM NaCl, 1 mM EDTA, 1 mM EGTA, 1% Triton, 0.1% sodium dodecyl sulfate, 2nM NaF (phosphatase inhibitor), and a protease inhibitor cocktail. Protein levels of the ER stress markers GRP78 and *Gadd*153, the autophagy markers LC3B, Atg4B, Atg5, Atg7, and p62, the autophagy signaling molecules PTEN, phosphorylated PTEN (p-PTEN) Akt, phosphorylated Akt (p-Akt), mTOR, phosphorylated mTOR (p-mTOR) were examined by standard Western immunoblotting. Membranes were probed with anti-GRP78 (1:1,000), anti-*Gadd*153 (1:1,000), anti-LC3B (1:1,000), anti-p62 (1:1,000), anti-Atg4B (1:1,000), anti-Atg5 (1:1,000), anti-Atg7 (1:1,000), anti-PTEN(1:1,000), anti-p-PTEN(Ser^380^1:1,000), anti-Akt (1:1,000), anti-p-Akt (Thr^473^, 1:1,000), anti-mTOR (1:1,000), anti-p-mTOR (Ser^2448^, 1:1,000), anti-p-ERK1/2 (Thr^202^/Tyr^204^, 1:1,000), anti-ERK1/2 (1:1,000), anti-p-SAPK/JNK (Thr^183^/Tyr^185^, 1:1,000), anti-SAPK/JNK (1:1,000), and anti-GAPDH (1:1,000, loading control) or anti-α-tubulin (1:1,000, loading control) antibodies. The anti-*Gadd*153 antibody was obtained from Santa Cruz Biotechnology (Santa Cruz, CA), the anti-p62 antibody was obtained from Progen biotechnik (Heidelberg, Germany) and all the other antibodies were purchased from Cell Signaling Technology (Beverly, MA). The membranes were incubated with horseradish peroxidase-coupled secondary antibodies. After immunoblotting, the film was scanned and intensity of immunoblot bands was detected with a Bio-Rad Calibrated Densitometer [23].

### Statistical analysis

Data were expressed as Mean ± SEM. Statistical significance (*p*<0.05) was estimated by one-way analysis of variation (ANOVA) followed by a Tukey’s test for *post hoc* analysis.

## Results

### General and echocardiographic properties of FVB and ADH mice with or without tunicamycin treatment

**Fig 1:**
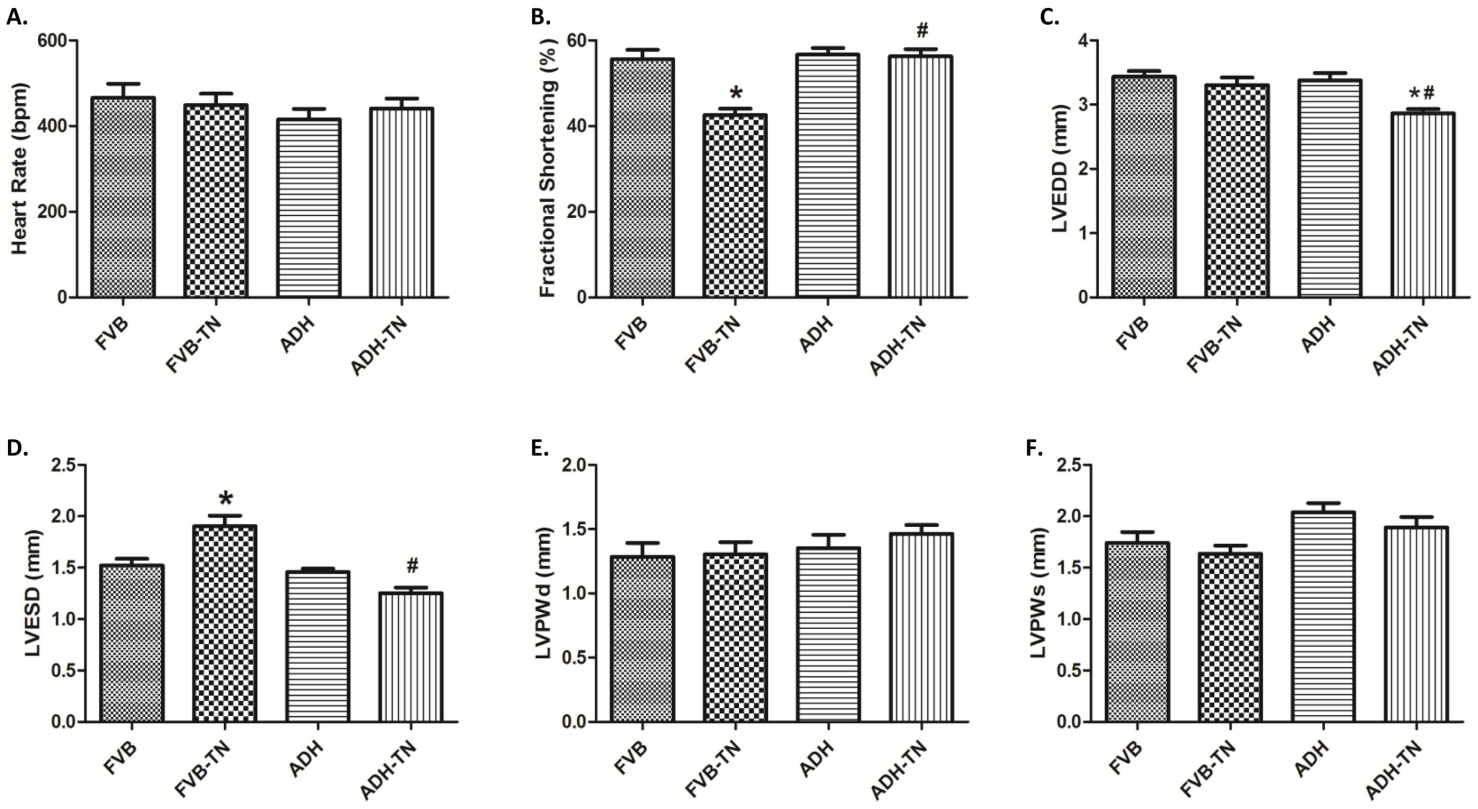
Effect of ADH transgene on tunicamycin (TN)-induced changes in echocardiographic properties. FVB and ADH mice were given the ER stress inducer TN (1 mg/kg, i.p., 48 hrs) prior to the assessment of echocardiographic properties. A: Heart Rate; B: Fractional shortening; C: LV end-diastolic diameter (LVEDD); D: LV end-systolic diameter (LVESD); E: LV posterior wall thickness at diastole (LVPWd); and F: LV posterior wall thickness at systole (LVPWs). Mean ± SEM; n = 7–9 mice per group, *p < 0.05 vs. FVB group, #p < 0.05 vs. FVB-TN group.

There was no difference in the body weight or the heart to body ratio in FVB and ADH mice with or without tunicamycin treatment (data not shown). Echocardiographic readings indicated that ER stress induction significantly increased LVESD and decreased fractional shortening, without affecting heart rate, LVEDD, LV posterior wall thickness during diastole (LVPWd) and systole (LVPWs). Although ADH overexpression itself failed to exert any significant changes on these echocardiographic parameters, it effectively mitigated tunicamycin-induced changes in LVESD and fractional shortening without affecting the pattern of responses for other indices. It is noteworthy that combined effects of tunicamycin and ADH transgene significantly reduced LVEDD compared to other groups (Fig 1).

### Mechanical and intracellular Ca^2+^ properties of cardiomyocytes

**Fig 2:**
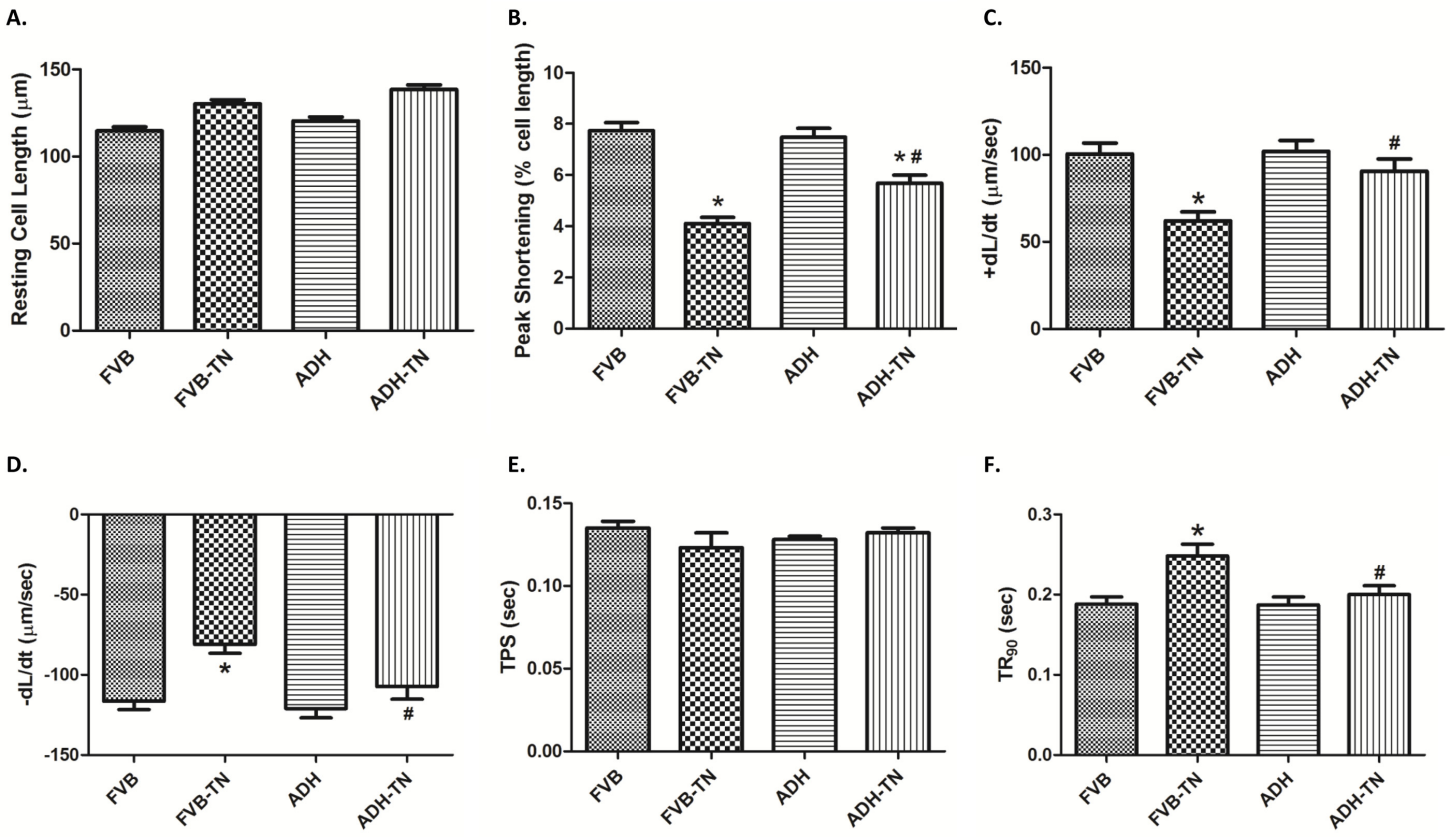
Effect of tunicamycin (TN) on cell shortening in isolated cardiomyocytes from FVB and ADH transgenic mice. A: Resting cell length; B: Peak shortening (% of resting cell length); C: Maximal velocity of shortening (+ dL/dt); D: Maximal velocity of relengthening (- dL/dt); E: Time-to-peak shortening (TPS); and F: Time-to-90% relengthening (TR_90_). Mean ± SEM, n = 105–107 cells per group, *p < 0.05 vs. FVB group; #p < 0.05 vs. FVB-TN group.

**Fig 3:**
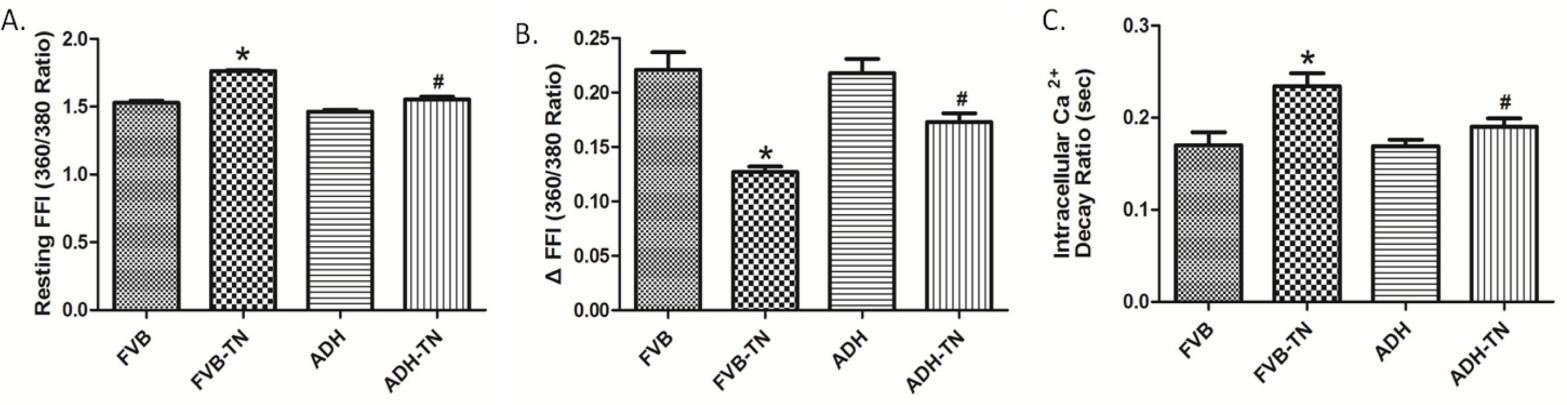
Effect of tunicamycin (TN) on intracellular Ca^2+^ properties in cardiomyocytes from FVB and ADH transgenic mice. A: Baseline Fura-2 fluorescence intensity (FFI); B: Electrically-stimulated increase in FFI (ΔFFI); and C: Intracellular Ca^2+^ decay rate. Mean ± SEM, n = 47–49 cells per group, *p < 0.05 vs. FVB group; #p < 0.05 vs. FVB-TN group.

Tunicamycin-induced ER stress significantly decreased peak shortening, maximal velocity of shortening and relengthening (± dL/dt) and prolonged time-to-90% relengthening (TR_90_) without affecting time-to-peak shortening (TPS), the effect of which was ablated by ADH transgene. ADH transgene itself failed to elicit any notable effect. Resting cell length tends to be greater for the two tunicamycin-treated groups although this phenomenon may simply be resulted from experimenter’s arbitrary choice of cell selection during cell recording (Fig 2). To explore the potential mechanism of actions involved in ADH-offered beneficial effect in cardiomyocyte mechanical responses, intracellular Ca^2+^ homeostasis was evaluated using the fluorescence dye fura-2. Our results indicated that tunicamycin significantly elevated baseline fura-2 fluorescence intensity (FFI), decreased electrically-stimulated rise of FFI (ΔFFI) and prolonged intracellular Ca^2+^ decay rate, the effects of which were nullified by ADH transgene (Fig 3). ADH transgene did not affect intracellular Ca^2+^ properties tested in the absence of tunicamycin challenge.

### Effect of ADH on tunicamycin-induced changes of ER stress

**Fig 4:**
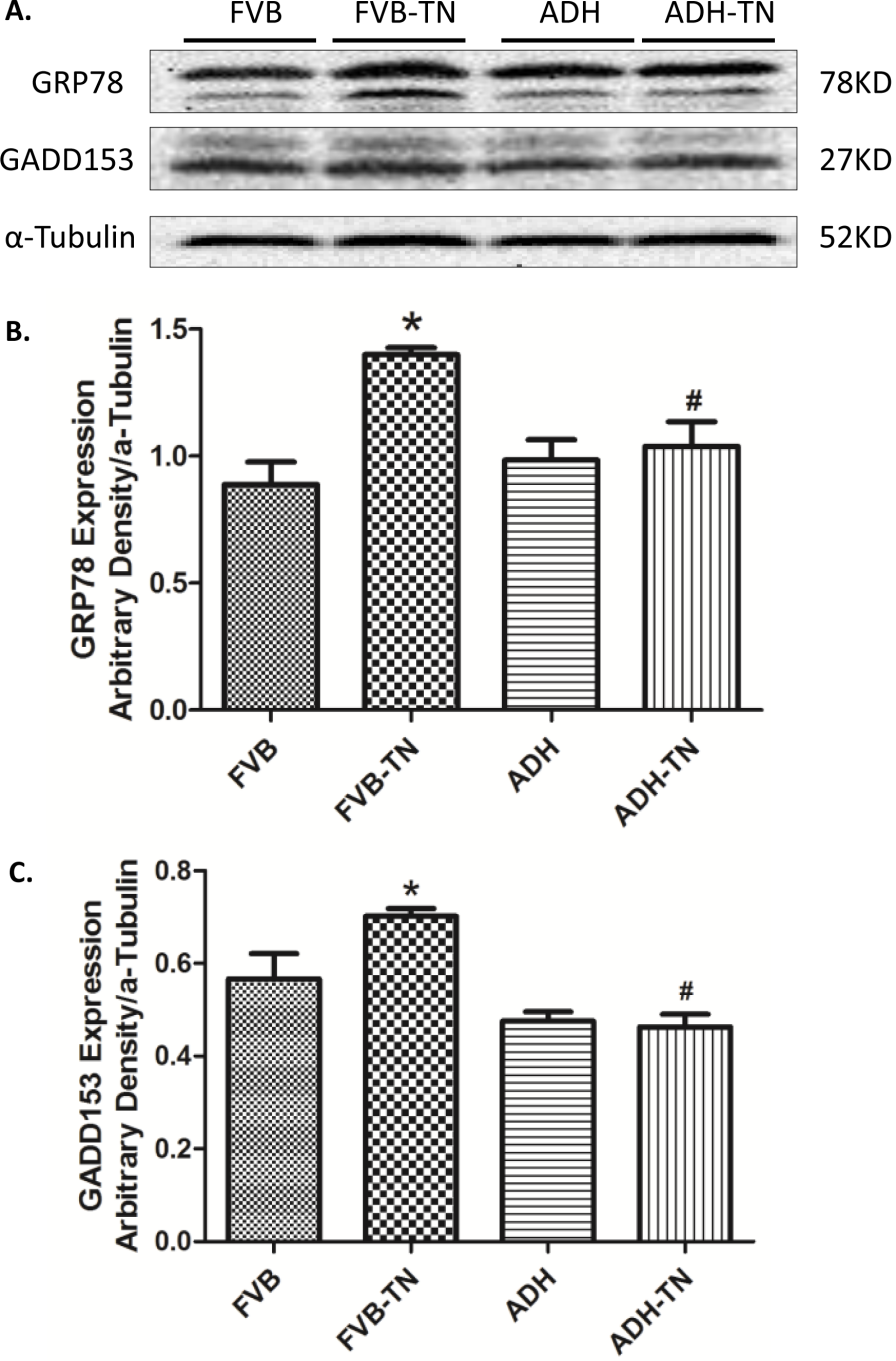
Effect of tunicamycin (TN) challenge (1 mg/kg, i.p.) on ER stress markers in FVB and ADH transgenic mice. A: Representative gel blots depicting expression of the ER stress markers GRP78, GADD153, and α-Tubulin (used as loading control); B: GRP78 expression; and C: GADD153 expression; All densities were normalized to respective α-Tubulin loading control. Mean ± SEM, n = 6–7 mice per group, *p < 0.05 vs. FVB group; #p < 0.05 vs. FVB-TN group.

Our results exhibited that tunicamycin significantly upregulated the expression levels of the ER stress markers GRP 78 and Gadd153, the effect of which was effectively obliterated by ADH transgene. The ADH transgene itself did not affect the levels of these ER stress markers (Fig 4).

### Effect of ADH on tunicamycin-induced oxidative stress

**Fig 5:**
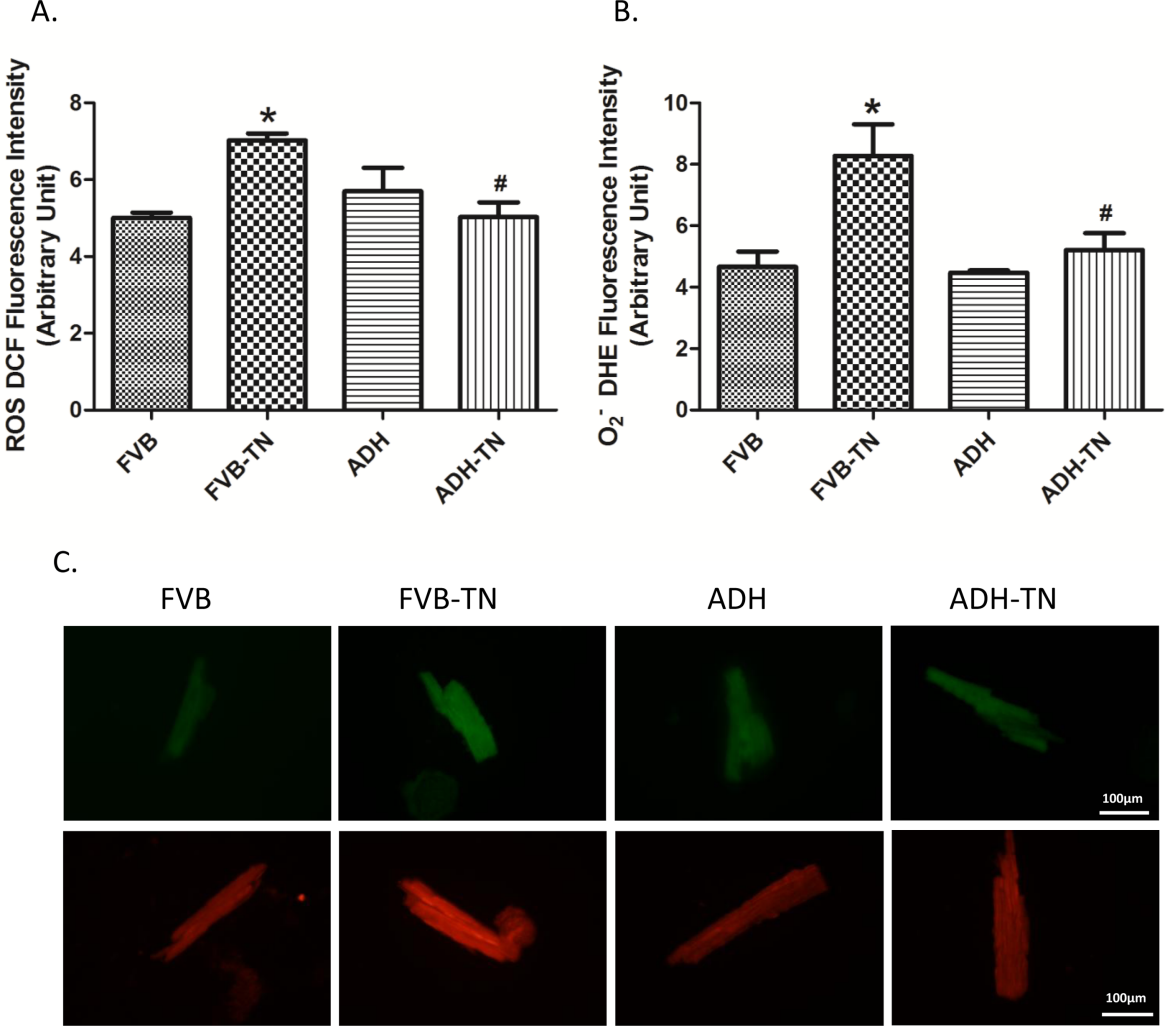
Assessment of oxidative stress using ROS and O_2_^-^ production in cardiomyocytes from FVB and ADH transgenic mice treated with or without tunicamycin (TN). A. ROS production assessed using DCF fluorescence; B. O_2_^-^ production assessed using DHE fluorescence; and C. Representative cell images of DCF (top panel) and DHE (lower panel) staining from FVB, FVB-TN, ADH, and ADH-TN groups. Mean ± SEM, n = 3 experiments per group, *p < 0.05 vs. FVB group, # p < 0.05 vs. FVB-TN group.

To explore possible mechanisms underlying the protective effect of ADH against tunicamycin-induced ER stress, oxidative stress status was examined using DCF and DHE fluorescence techniques. DCF staining is widely used for detection of reactive oxygen species (ROS), while DHE is a specific O_2_^-^ indicator. Data from both DCF and DHE staining showed that tunicamycin promoted oxidative stress. Interestingly, ADH alleviated tunicamycin-induced oxidative stress without eliciting any effect by itself (Fig 5).

### Effect of ADH on tunicamycin-induced changes of autophagy and relative signaling pathway markers

**Fig 6:**
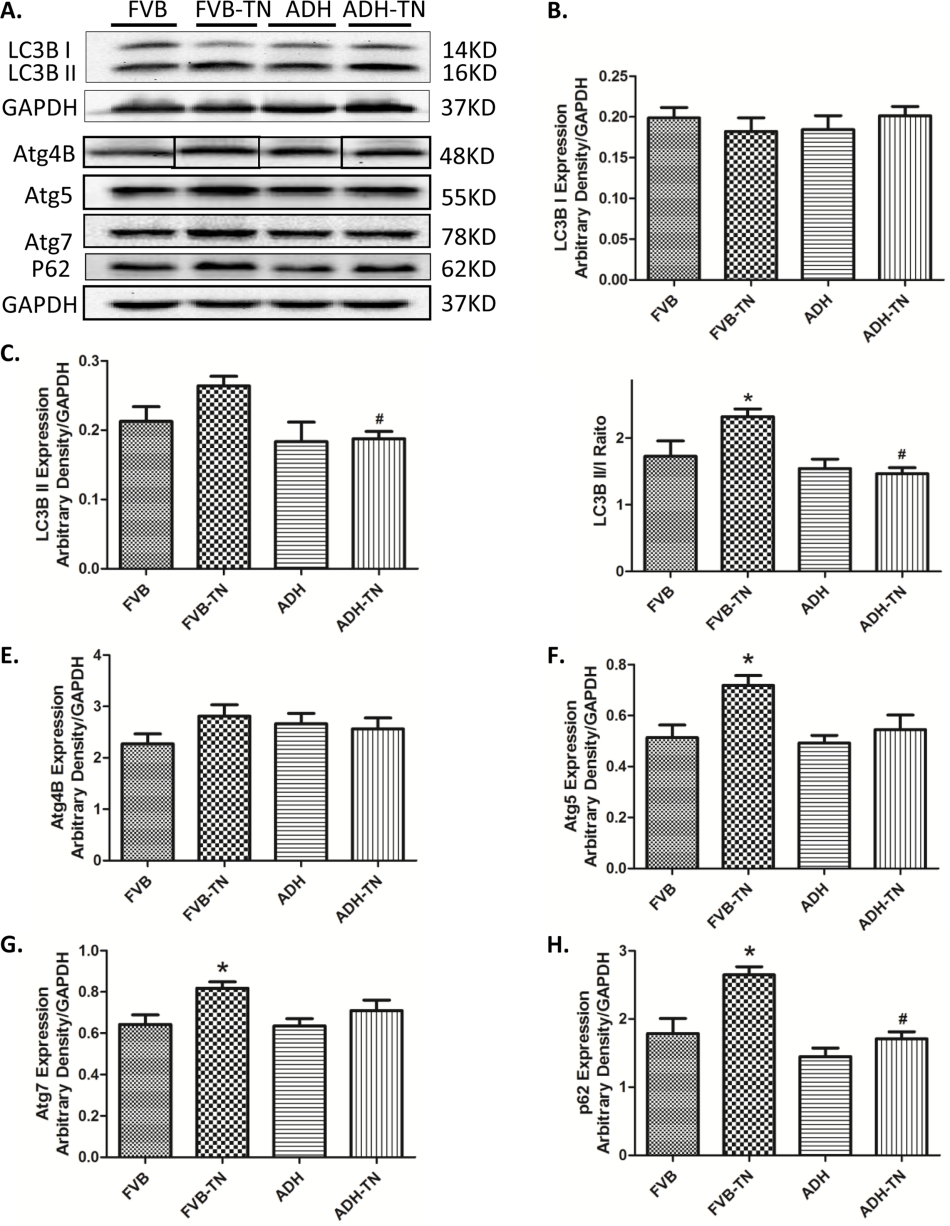
Effect of tunicamycin (TN) challenge (1 mg/kg, i.p.) on autophagy markers in FVB and ADH mice. A: Representative gel blots depicting expression of LC3B, Atg4B, Atg5, Atg7 and P62 and GAPDH (used as loading control); B: LC3B-I expression; C: LC3B-II expression; D: LC3B-II-to-LC3B-I ratio; E: Atg4B expression; F: Atg5 expression; G: Atg7 expression; and H: P62 expression. All densities were normalized to that of GAPDH. Mean ± SEM, n = 6–7 mice per group, *p < 0.05 vs. FVB group; #p < 0.05 vs. FVB-TN group.

**Fig 7:**
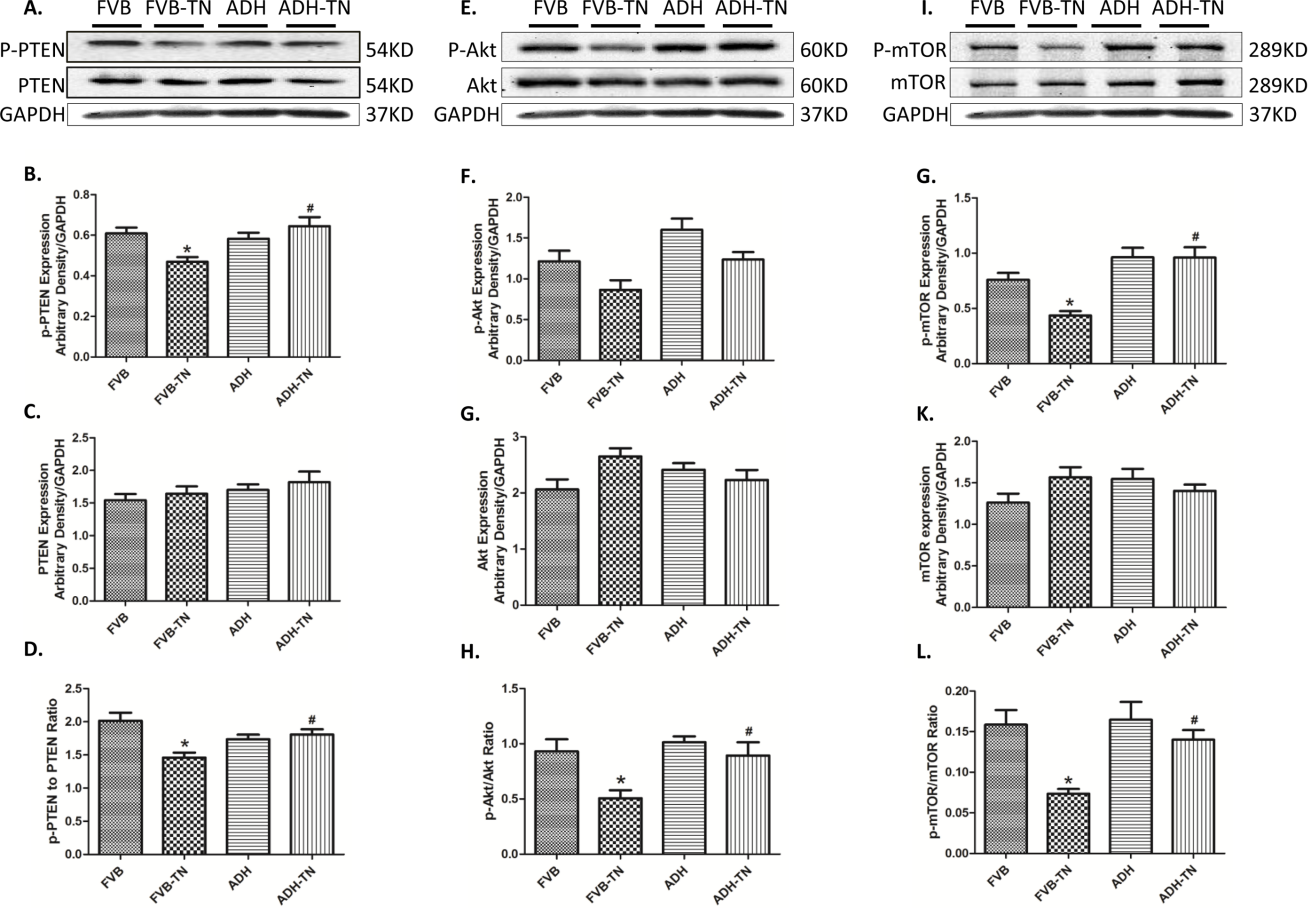
Effect of tunicamycin (TN) challenge (1 mg/kg, i.p.) on related autophagic signaling proteins in FVB and ADH mice. A, E, I: Representative gel blots depicting expression of PTEN, phosphorylated PTEN, Akt, phosphorylated Akt, mTOR, phosphorylated mTOR, and GAPDH (used as loading control); B: Phosphorylated PTEN expression; C: PTEN expression; D: p-PTEN-to-PTEN ratio; F: Phosphorylated Akt expression; G: Akt expression; H: p-Akt-to-Akt ratio; J: Phosphorylated mTOR expression; K: mTOR expression; and L: p-mTOR-to-mTOR ratio. All densities were normalized to that of GAPDH. Mean ± SEM, n = 6–7 mice per group, *p < 0.05 vs. FVB group; #p < 0.05 vs. FVB-TN group.

**Fig 8:**
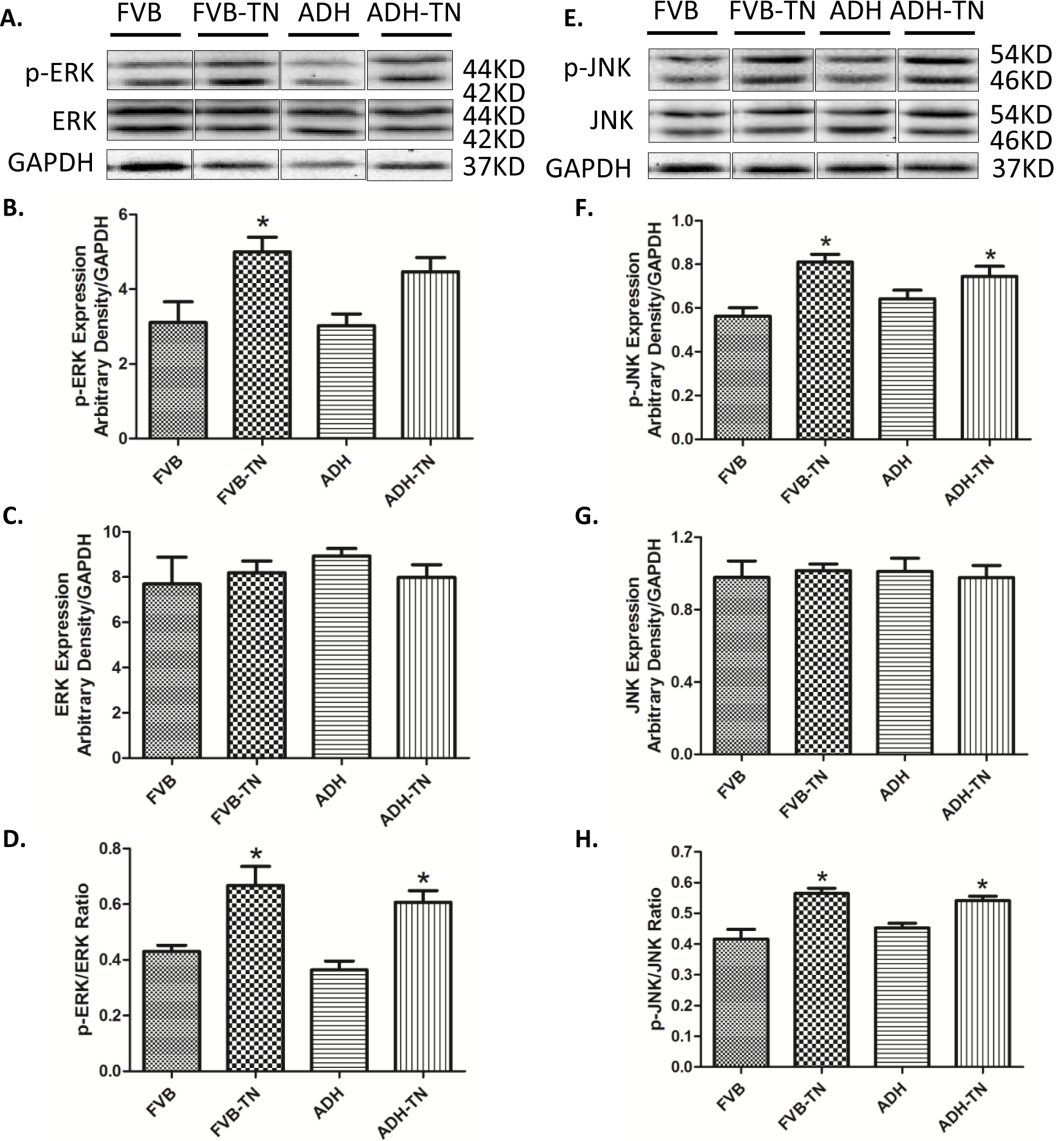
Effect of tunicamycin (TN) challenge (1 mg/kg, i.p.) on ERK-JNK pathway signaling proteins in FVB and ADH transgenic mice. A, E: Representative gel blots depicting expression of ERK, phosphorylated ERK, JNK, phosphorylated JNK, and GAPDH (used as loading control); B: Phosphorylated ERK expression; C: ERK expression; D: p-ERK-to-ERK ratio; F: Phosphorylated JNK expression; G: JNK expression; and H: p-JNK-to-JNK ratio. All densities were normalized to that of the loading control GAPDH. Mean ± SEM, n = 6 mice per group, *p < 0.05 vs. FVB group; #p < 0.05 vs. FVB-TN group.

To further explore mechanisms of action involved in the protective role of ADH enzyme against tunicamycin, levels of the autophagy markers LC3B, Atg4B, Atg5, Atg7 and P62 were examined in FVB and ADH transgenic mice with or without tunicamycin challenge. Our results revealed that tunicamycin significantly upregulated LC3B II, LC3BII/I ratio, Atg5, Atg7 and p62 (but not Atg4B) expression. ADH ablated or significantly attenuated tunicamycin-induced upregulation in LC3B II, LC3BII/I ratio, Atg5, Atg7 and p62 levels. ADH itself did not affect levels of these autophagy markers tested (Fig 6). To explore the signaling mechanisms behind ADH-offered protection against ER stress-elicited autophagy, the autophagic signaling proteins including pan and phosphorylated PTEN, Akt, mTOR, ERK and JNK were examined. Our results indicated that ER stress induction significantly decreased phosphorylation of PTEN, Akt and mTOR, without affecting pan protein expression of PTEN, Akt and mTOR. ADH transgene significantly restored the phosphorylation level of PTEN, Akt and mTOR in the face of tunicamycin treatment, without influencing the total expression of PTEN, Akt and mTOR (Fig 7). In addition, ER stress significantly increased phosphorylation of ERK and JNK, without obviously influencing pan protein expression of ERK and JNK, the response pattern was unaffected by ADH transgene (with the exception of absolute phosphorylation level of ERK). ADH transgene itself did not affect levels of pan or phosphorylated ERK and JNK (Fig 8).

### Effect of autophagy and ADH as well as inhibition of autophagy, Akt and mTOR on ER stress-induced cardiomyocyte dysfunction

**Fig 9:**
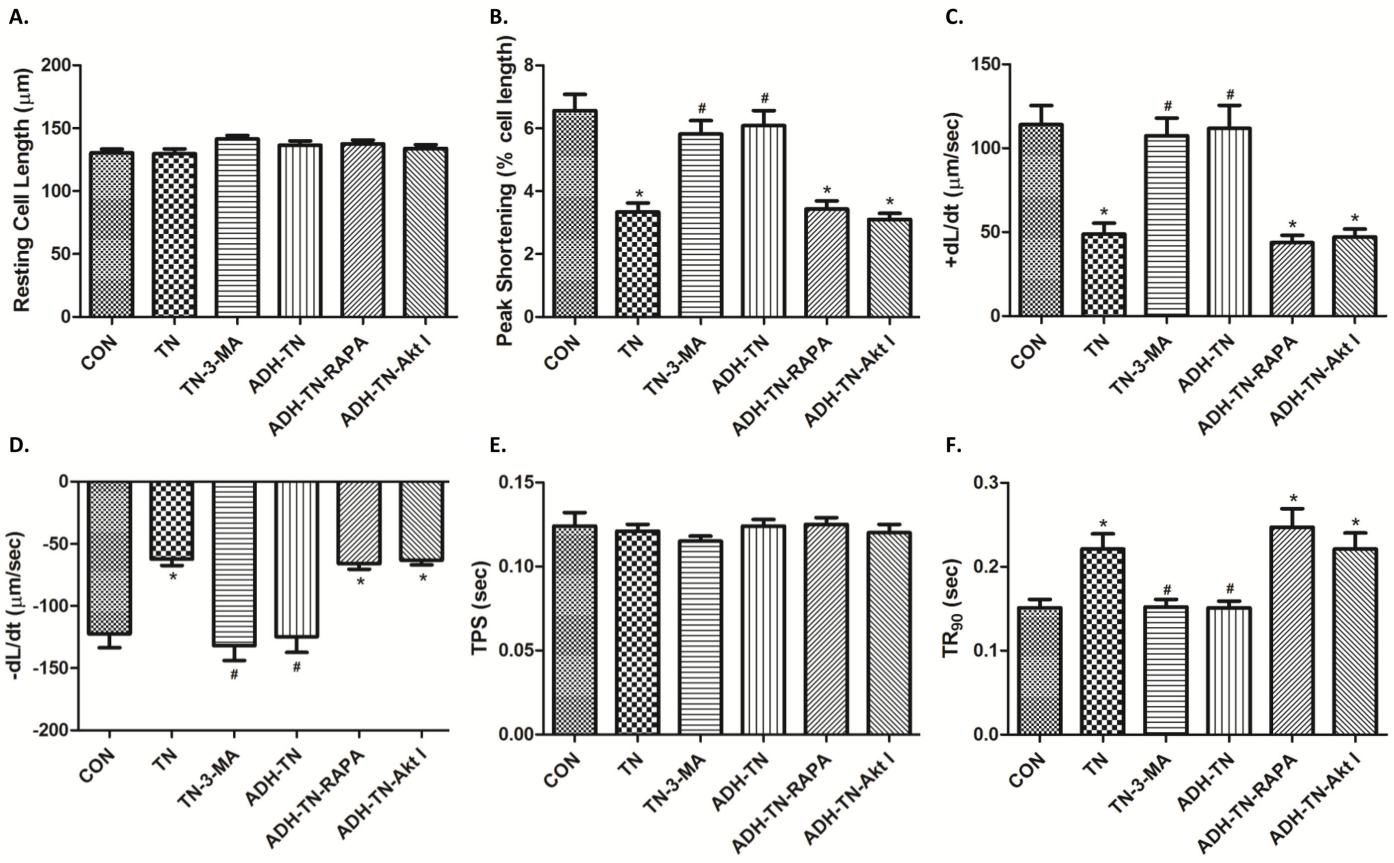
Role of autophagy, Akt and mTOR in tunicamycin (TN)-induced cardiomyocyte contractile responses. Isolated cardiomyocytes from FVB or ADH mice were incubated with TN (3 μg/ml) for 4 hrs in vitro prior to assessment of mechanical properties. A cohort of FVB or ADH cardiomyocytes was co-incubated in the presence of the autophagy inhibitor 3-methyladenine (3-MA, 3 mM), the Akt inhibitor AktI (1 μM) or the mTOR inhibitor rapamycin (5 μM) with or without TN. A: Resting cell length; B: Peak shortening (% of resting cell length); (C): Maximal velocity of shortening (+ dL/dt); (D): Maximal velocity of relengthening (- dL/dt); (E): Time-to-peak shortening (TPS); and (F): Time-to-90% relengthening (TR_90_). Mean ± SEM, n = 105–107 cells per group, *p < 0.05 vs. FVB group; #p < 0.05 vs. FVB-TN group.

We further performed *in vitro* study to examine 1) the effect of tunicamycin on cardiomyocyte contractile function in the absence or presence of the autophagosome formation inhibitor 3-methyladenine (3-MA, 3 mM) prior to assessment of mechanical properties. 2) The effect of ADH on tunicamycin-treated cardiomyocyte contractile function in the absence or presence of the Akt inhibitor AktI (1 μM) or the mTOR inhibitor rapamycin (5 μM). Our observations revealed that tunicamycin exposure *in vitro* significantly decreased peak shortening, ±dL/dt and prolonged TR_90_ without affecting TPS, the effect of which was abolished by the autophagy inhibitor 3-MA and ADH. However, the protective effect of ADH was negated by the Akt inhibitor Akt I and the mTOR inhibitor rapamycin (Fig 9).

## Discussion

Our results revealed for the first time that ADH transgene protected against tunicamycin-induced cardiac dysfunction through attenuation of ER stress, oxidative stress, and PTEN-Akt-mTOR regulated autophagy. Our findings revealed that tunicamycin impaired echocardiographic and cardiomyocyte contractile properties, and intracellular Ca^2+^ handling in association with ROS accumulation, ER stress and excessive autophagy. Intriguingly, these ER stress-induced changes in mechanical and stress signaling properties were mitigated by cardiac-specific overexpression of ADH, suggesting the therapeutic potential of ER stress in cardiovascular diseases.

### Tunicamycin-induced abnormalities in ROS, autophagy and cardiac function

ER stress, as triggered by tunicamycin in our current model [36], plays a key role in the onset and development of cardiovascular diseases [1, 9, 37, 38]. GRP78 is a major ER chaperone, which is regarded to be an important marker for both unfolded protein response (UPR) and ER stress [39]. Gadd 153 is a key component of the UPR signaling [39]. From our data, tunicamycin-induced ER stress is evidenced by elevated expression of GRP78 and Gadd153. Data from our current study revealed increased LVEDD and LVESD, reduced fractional shortening, reduced peak shortening, decreased maximal velocity of shortening/relengthening, and prolonged relengthening duration as well as elevated resting intracellular Ca^2+^ levels, decreased electrical-stimulated intracellular Ca^2+^ rise and delayed intracellular Ca^2+^ clearance following short-term tunicamycin exposure, in line with earlier studies [23, 24]. In our hands, combined effect of ER stress and ADH transgene overtly decreased LVEDD. Although the precise mechanism behind this drop in LVEDD in the ADH-TN group is unknown, it may be related to possible interaction between ER stress and ADH on cardiac remodeling process or simply to a decreased venous return (smaller preload). One likely explanation for tunicamycin-induced myocardial damage is through oxidative stress due to ROS and O_2_^-^ accumulation as observed in our current study. Elevated ROS level was reported in tunicamycin-treated murine model from our earlier study [24]. High level of ROS may trigger ER stress. On the other hand, ER stress may promote oxidative stress/ROS generation by perturbing mitochondrial function [40].

ER stress is known to induce autophagy in various pathological conditions, such as high fat diet-induced metabolic disorders [41], and diabetes [42], possibly via the IRE 1/JNK/p38 pathway and ATF4 dependent activation [43]. Although autophagy induction may be protective by removing the damaged proteins and organelles, excessive autophagy is detrimental for cardiomyocyte survival as observed in our short-term tunicamycin challenge model. Elevated autophagy marker LC3B-II, Atg5, Atg7 and the autophagy adaptor protein p62 were noted in response to tunicamycin challenge. The main function of Atg4 is to cleave LC3 to LC3-I, among which Atg4B is the most efficient isoform [44]. In our study, tunicamycin failed to significantly affect the level of Atg4B, indicating tunicamycin has little effect for preparation of LC3-I. Elevated autophagy induced by tunicamycin is also supported by our *in vitro* finding where autophagy inhibition using 3-MA (which can suppress the early-stage autophagosome formation) reversed tunicamycin-induced cardiomyocyte dysfunction, confirming the role of autophagy in ER stress-induced cardiac dysfunction. Our *in vitro* findings further revealed that inhibition of Akt and mTOR may nullify ADH-offered cardioprotective effect against ER stress, suggesting an important role for PTEN-Akt-mTOR-regulated autophagy in tunicamycin- and ADH-induced cardiac responses. It is noteworthy that p62 levels were higher alongside with LC3B-II levels in response to ER stress. This seems to be puzzling for increased autophagy. p62 acts as an adaptor to symbolize the lysosomal degradation/clearance efficiency. Typically, p62 should be lower with a good function of autophagy and autophagy flux function. However, with overwhelmed autophagy in the setting of ER stress, the newly formed autophagosomes may be too much for the lysosomes to clear, resulting in a transient accumulation of this autophagy cargo protein.

Our data revealed that tunicamycin treatment may decrease phosphorylation of PTEN and its downstream signaling molecules Akt and mTOR, two pivotal inhibitors of autophagy. PTEN is a suppressor of PI3K and Akt. PTEN activity can be negatively regulated by phosphorylation in an post-translational modification manner, leading to inactivation of PTEN [45]. In our hands, PTEN phosphorylation was suppressed by tunicamycin, leading to the inhibition of PI3K-Akt-mTOR signaling (less inactive form of PTEN). This is in concert with reduction in phosphorylated Akt and mTOR levels following tunicamycin treatment. Acetaldehydes, similar to 4-HNE and other ROS products, may influence PTEN phosphorylation. Our earlier study showed that 4-HNE treatment significantly suppressed PTEN phosphorylation [46]. Given that tunicamycin can lead to ROS accumulation, it is likely that tunicamycin may suppress PTEN phosphorylation through ROS accumulation. Another interesting finding in our study is that tunicamycin treatment may promote phosphorylation of the stress signaling kinases ERK and JNK, suggesting a possible role for these stress signaling cascades in autophagy induction. JNK is known to participate in autophagic events induced by stress conditions, including oxidative stress [47] and ER stress [48]. ERK/JNK is believed to regulate autophagy through Beclin-1 and FoxO1-dependent ATG gene regulation [49]. However, our experimental findings did not favor a major role for ERK and JNK signaling in ADH transgene-offered protection against ER stress-induced induction of autophagy.

### ADH-offered protection against tunicamycin-induced abnormalities

Probably the most intriguing finding from our study was the cardioprotective property of ADH against ER stress. ADH was reported to augment ethanol-induced cardiac contractile anomaly [16, 50] although our current finding suggests that ADH is capable of attenuating tunicamycin-induced rises in ER stress, ROS, and autophagy. Earlier evidence suggested that Class I and IV ADH may reduce endogenous aldehyde and 4-HNE [18, 19]. Our experimental findings revealed that ADH attenuated tunicamycin-induced ER stress in concert with the changes in ROS production. Although the precise mechanism of action behind ADH-offered protection against tunicamycin-induced autophagy induction is unknown at this time, two mechanisms may be considered. First, ER stress is known to impair cardiac function through inducing autophagy. It was also proved by our *in vitro* study that inducing autophagy by inhibition of Akt and mTOR abolished the ADH-offered protection against tunicamycin. Second, restored PTEN signaling may play a role in ADH-offered inhibition of autophagy. Given that ROS and its product 4-HNE may suppress PTEN phosphorylation, ADH may restore PTEN phosphorylation level by suppression of ROS. Restored PTEN phosphorylation leads to the activation of the PTEN downstream signaling Akt-mTOR cascade, leading to suppressed autophagy. Inhibition of Akt or mTOR diminished ADH-offered protective effect against tunicamycin according to the in vitro study. As mentioned earlier, ERK/JNK signaling pathway is unlikely to be involved in ADH-offered protection in autophagy, and possibly mechanical benefit under ER stress. Taken together, these findings favored an important role of PTEN-Akt-mTOR pathway in ADH transgene-offered protection of tunicamycin-induced excessive autophagy.

**Fig 10:**
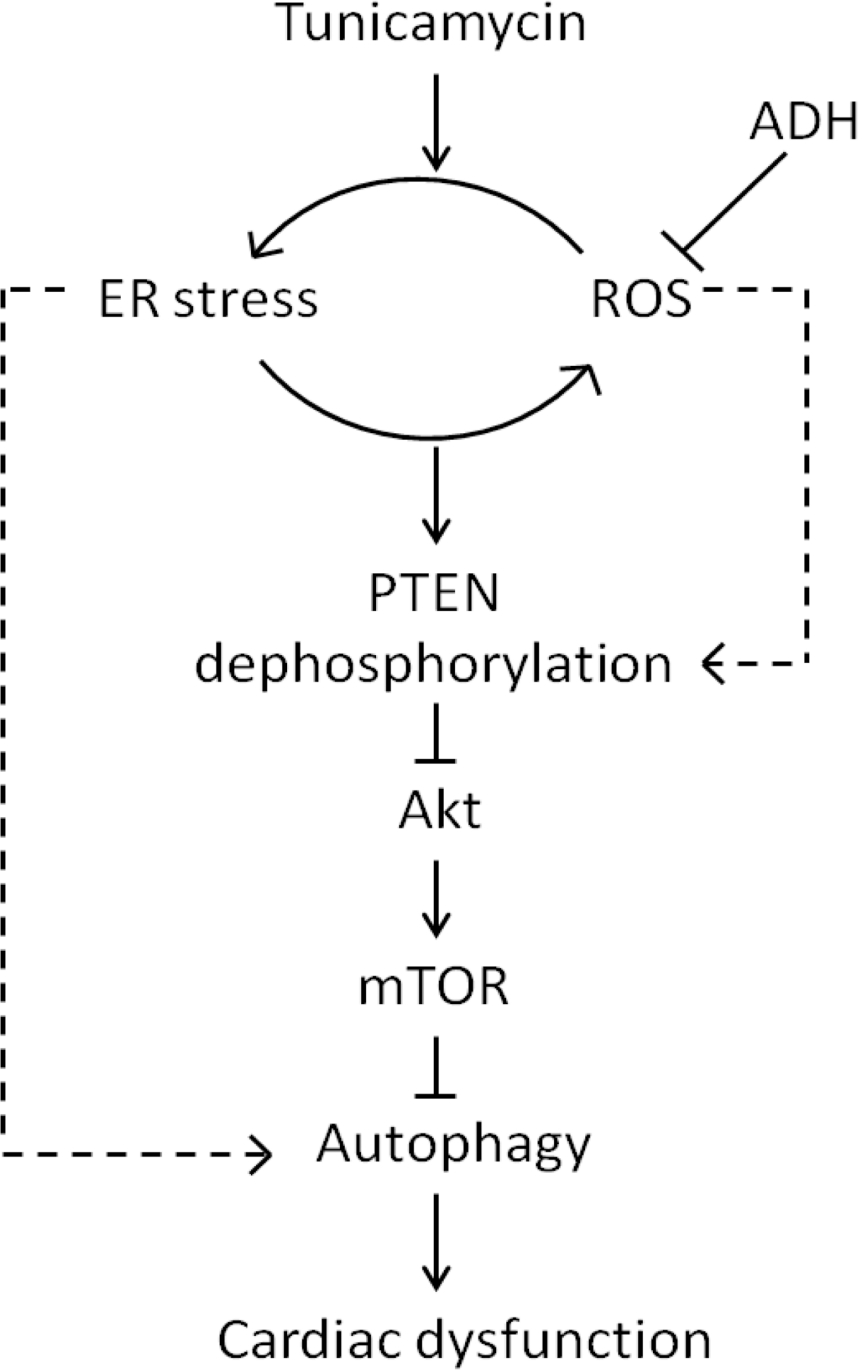
Schematic diagram depicting the proposed signaling mechanism behind tunicamycin- and ADH transgene-induced myocardial contractile responses.

ER stress serves as a major culprit factor for cardiovascular diseases. On the other hand, ADH genetic polymorphism influences ADH enzymatic activity, the prevalence, pathogenesis and clinical outcome of cardiovascular diseases. Finding from our current study suggests that ADH overexpression may preserve the heart against ER stress and ROS in association with alleviating PTEN-Akt-mTOR-regulated autophagy (Fig 10). Although our data may shed some light towards the interplay among ER stress, ROS, autophagy and ADH enzymatic activity in cardiac pathophysiology, the precise mechanism(s) of action behind ADH overexpression-and ER stress-mediated cardiac responses warrants further scrutiny.
