# Expression of Concern: Alcohol Dehydrogenase Protects against Endoplasmic Reticulum Stress-Induced Myocardial Contractile Dysfunction via Attenuation of Oxidative Stress and Autophagy: Role of PTEN-Akt-mTOR Signaling

**DOI:** 10.1371/journal.pone.0353814

**Published:** 2026-07-15

**Authors:** The PLOS One Editors

The *PLOS One* Editors issue this Expression of Concern to inform readers that the articles cited as References 7, 23, 25, 27, 33, 38, and 50 were retracted after [1] was published. Readers are advised to interpret the article [1] with caution.

The first author stated that all cited information from the retracted references is common knowledge or standard methodology, or corroborated by other references.

## References

[1] Pang J, Fuller ND, Hu N, Barton LA, Henion JM, Guo R, et al. Alcohol Dehydrogenase Protects against Endoplasmic Reticulum Stress-Induced Myocardial Contractile Dysfunction via Attenuation of Oxidative Stress and Autophagy: Role of PTEN-Akt-mTOR Signaling. PLoS One. 2016;11(1):e0147322. doi: 10.1371/journal.pone.0147322 26807981 PMC4726758

